# Non-medial Subperiosteal Abscess in a 63-Year-Old Female: A Case Report and Literature Review

**DOI:** 10.7759/cureus.43843

**Published:** 2023-08-21

**Authors:** Elaf S Alabdulrazzaq, Mohammed N Alajmi, Ahmed S Alsalem

**Affiliations:** 1 Ophthalmology, Prince Sultan Military Medical City, Riyadh, SAU

**Keywords:** proptosis, chemosis, sinusitis, loss of vision, sub-periosteal abscess, orbital cellulitis

## Abstract

We present a rare case of non-medial subperiosteal abscess secondary to orbital cellulitis in a 63-year-old female. The patient reported a five-day history of progressive swelling, pain, and diminished vision in the left eye. Computed tomography (CT) of the orbit revealed an extraconal soft tissue density, suggestive of an orbital collection, which when correlated clinically and radiologically, was diagnosed as orbital cellulitis secondary to sinusitis, leading to a subperiosteal abscess. Despite undergoing multiple external drainage procedures, the patient, unfortunately, experienced complete vision loss in the affected eye. While non-medial abscesses due to orbital cellulitis are infrequent, they are often associated with more severe outcomes, including vision loss and intracranial complications, compared to those in medial locations. This case underscores the importance of combined surgical approaches, including both sinus and external drainage, to prevent severe vision loss and potentially life-threatening intracranial sequelae.

## Introduction

Orbital cellulitis is a critical ocular emergency that can precipitate life-threatening complications, including meningitis, cerebral venous thrombosis, and brain abscess and empyema [1]. It is characterized as an infection of the ocular adnexa and tissues situated posterior to the orbital septum. The orbital septum serves as a delineating barrier, differentiating the less severe preseptal cellulitis from the more critical, potentially life-threatening orbital cellulitis [2].

The primary etiological factor for orbital cellulitis is an infection of the paranasal sinuses, predominantly originating from the ethmoid, followed by maxillary and frontal sinuses. Nonetheless, orbital cellulitis can emerge due to the progression of preseptal cellulitis or more frequently through the following pathways: 1) direct transmission or infection from sources such as insect bites or trauma; 2) infection dissemination from adjacent ocular and periocular structures, for instance, sinusitis, dacryocystitis, and hordeolum; and 3) hematogenous spread stemming from conditions like otitis media, pneumonia, and sepsis [2]. Inadequate diabetes control is a known predisposing factor for the particularly devastating mucormycosis variant of orbital cellulitis, which can manifest with multiple cranial nerve palsies, termed “orbital apex syndrome” [3]. Notably, mucormycosis can also afflict immunocompetent individuals, as documented by Badiee et al. in a case involving a healthy two-year-old boy [4].

In light of these findings, we detail a case involving an adult female who presented with painful proptosis. Comprehensive history, examination, and investigative data collectively confirmed a diagnosis of orbital cellulitis, which was further characterized by an atypical location and presentation of a subperiosteal abscess. This report strictly adheres to the ethical principles delineated in the Helsinki Declaration.

## Case presentation

A 63-year-old Saudi female, with a history of type 2 diabetes mellitus and hypothyroidism on treatment, presented to the Prince Sultan Military Medical City in Riyadh with five days of progressive, painful proptosis of the left eye. The patient denied a history of trauma, insect bites, topical medication use, or similar prior episodes. She also had no recent dental procedures or past ocular inflammatory conditions. Nevertheless, she did report a remote history of allergic rhinitis.

The initial ophthalmic examination results are detailed in Table 1.

**Table 1:** Initial ophthalmic examination

Site	OD	OS
Visual acuity	20/20	20/50
Intra-ocular pressure	14	14
Pupils	Round and reactive, No afferent pupillary defect (APD)	Round and reactive, No afferent pupillary defect
External eye exam	Within normal limits	Periorbital erythematous, tender swelling; no identifiable localized swelling such as chalazion or stye; no signs of dacryocystitis
Extra-ocular motility	Full in all gazes	Restricted motility: -4 abduction deficit, -2 adduction, supraduction, and infraduction
Lids/lacrimal	Within normal limits	Inflamed swollen lacrimal gland; otherwise within normal limits and anatomical contour
Conjunctiva/sclera	Clear and white	Extensive chemosis with mild injection
Cornea	Clear	Clear
Anterior chamber	Deep and quiet	Deep and quiet
Iris	Round and regular	Round and regular
Lens	Clear	Clear
Fundus	Healthy disc	Healthy disc, no swelling

**Figure 1:**
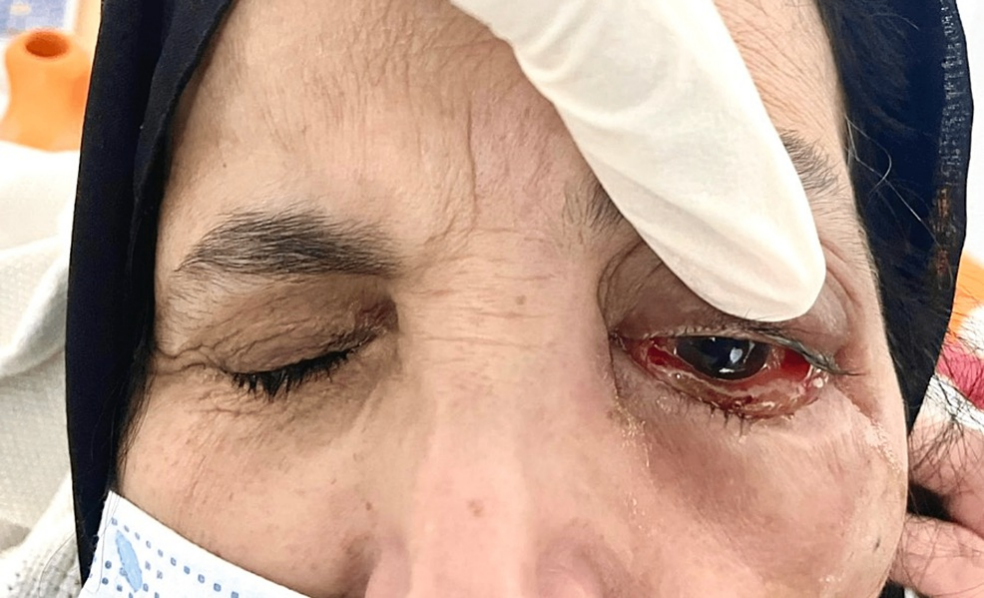
Gross image of the patient’s presentation, depicting a swollen, erythematous left eye and the periocular region extending inferiorly to the cheek, accompanied by notable conjunctival chemosis

Vital signs indicated that the patient was stable and afebrile, as depicted in Figure 1.

Laboratory investigations, which included conjunctival and nasal swabs and a sepsis workup, revealed negative blood, nasal, and urinary cultures. However, there was leukocytosis with a white blood cell count of 11.9 and a positive conjunctival swab yielding methicillin-sensitive Staphylococcus aureus.

**Figure 2:**
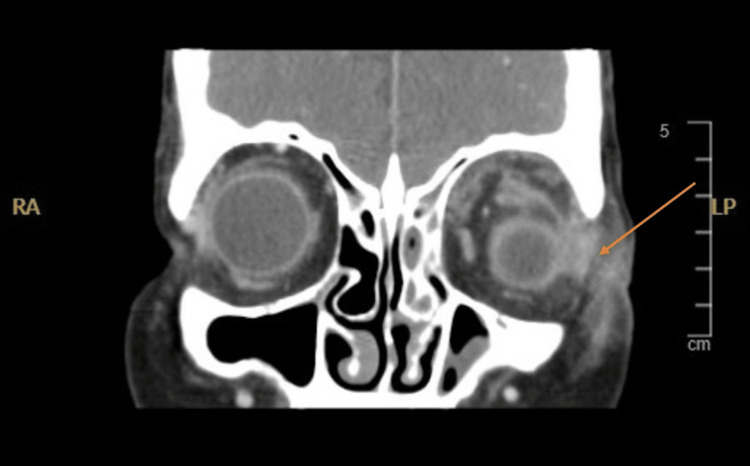
Enhanced coronal CT scan of the brain and orbit reveals opacified left pan-sinuses and mild pacification of right paranasal sinuses (including maxillary, frontal, and ethmoid air cells) and an enhanced collection in the left orbital region (arrow) along with the lacrimal gland

A contrast-enhanced CT scan of the orbit and brain demonstrated left eye proptosis and increased enhancement of the left lacrimal gland. There was also a small collection measuring 0.5 × 0.6 cm and an extraconal lesion of soft tissue density with internal air foci measuring 1.2 × 1.6 cm. The optic nerve sheath was unremarkable. The findings suggested a probable infectious process, although an extraconal underlying mass could not be ruled out. The CT further revealed opacification of the left paranasal sinuses involving the maxillary, frontal, and ethmoid air cells, with mild opacification of the right paranasal sinuses. There was no evidence of cerebral venous thrombosis or meningeal enhancement, as illustrated in Figure 2.

Urgent consultations with otorhinolaryngology (ENT) and infectious disease (ID) specialists were sought. The patient was started on intravenous broad-spectrum antibiotics and was monitored every six hours. The ENT assessment on nasal endoscopy showed a patent nasal cavity and patent nasopharynx with no apparent polyps or masses. Moreover, they recommended conservative management with daily assessments and did not advise any surgical intervention at that juncture.

**Figure 3:**
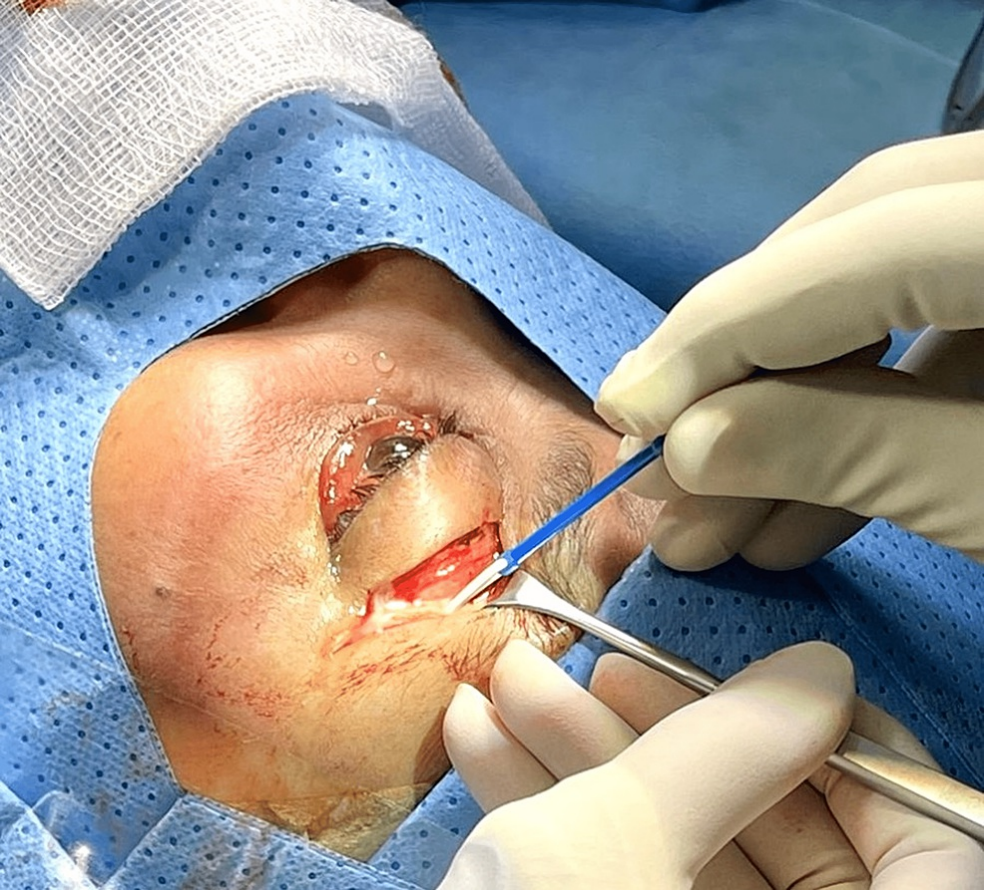
Gross image taken during the orbital exploration and drainage procedure of the left orbital abscess

On subsequent evaluation, the patient showed progression despite antibiotic therapy. Ophthalmic findings included a visual acuity limited to counting fingers, a 2+ afferent pupillary defect, and an exacerbation of periorbital and ocular soft tissue inflammation, manifested as pronounced swelling, severe pain, and a near-immobile globe with negligible motility. The patient underwent an emergency orbital exploration and drainage of the orbital abscess (Figure 3).

Specimens were collected for culture and staining from the drained abscess. Postoperatively, the patient was monitored every six hours and underwent a follow-up MRI with contrast.

**Figure 4:**
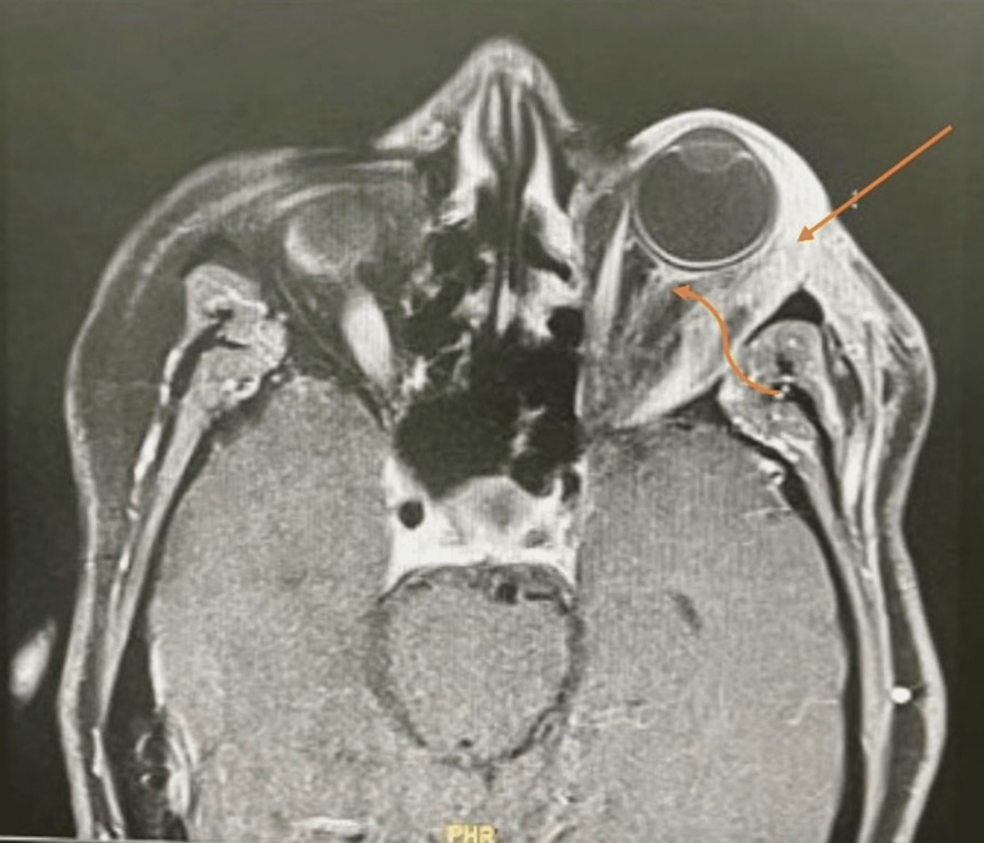
Postoperative MRI of the brain and orbit displays a residual left extraconal enhancing collection (straight arrow) This is associated with the progression of both preseptal and post-septal inflammatory changes as well as inflammation in the left optic nerve sheath (curved arrow), leading to secondary scleritis.

The postoperative assessment revealed that the patient’s condition had deteriorated compared to the preoperative status. The MRI, depicted in Figure 4, revealed a residual left extraconal peripherally enhancing collection associated with a mild progression of preseptal and post-septal inflammatory changes or cellulitis.

Further findings included inflammation of the left optic nerve sheath, secondary scleritis, and conjunctivitis. There was stable proptosis of the left eye and secondary bilateral frontal and left temporal pachymeningeal enhancement. The clinical examination findings are presented in Table 2.

**Table 2:** Post-abscess drainage ophthalmological examination

Site	OD	OS
Visual acuity	20/20	Poor light perception (LP)
Intra-ocular pressure	16	14
Pupils	Round and reactive, No afferent pupillary defect	3+ afferent pupillary defect
External eye exam	Within normal limits	Severe tender periorbital erythematous swelling (worse than pre-op status)
Extra-ocular motility	Full in all gazes	Restricted, resembling a “Fixed Frozen Globe”
Lids/lacrimal	Within normal limits	Inflamed, swollen lacrimal gland; otherwise within normal limits
Conjunctiva/sclera	Clear and white	Extensive chemosis with injection
Cornea	Clear	Clear
Anterior chamber	Deep and quiet	Deep and quiet
Iris	Round and regular	Round and regular
Lens	Clear	Clear
Fundus	Healthy disc	Hyperemic disc

A CT scan of the facial bones and sinuses revealed inflammatory changes within the paranasal sinuses, accompanied by multiple air pockets and opacification of the frontal, maxillary, and ethmoid sinuses. Notably, there was no evidence of sinonasal osseous destruction.

An urgent re-consultation with the ENT department was sought for the potential combined functional endoscopic sinus surgery (FESS) and orbital abscess evacuation. However, the recommendation was against intervention, given the inaccessibility of the most inflamed sinus. Subsequently, the patient underwent another urgent drainage of the orbital abscess. During this procedure, specimens were again collected for staining, culture, and histopathological analysis. Postoperatively, a reduction in swelling and pain was noted while VA and pupil reactions remained stable at LP with poor projection and an APD of 3+.

A follow-up MRI after drainage demonstrated interval resolution and a decrease in the size of the left eye globe collection, albeit with a small residual component. There was an interval reduction in inflammatory changes in the intraconal, conal, extraconal, and preseptal regions. Changes in the left orbital roof bone marrow were suggestive of osteomyelitis, and there was interval regression of inflammation in the frontal and ethmoid sinuses.

The patient’s treatment regimen continued to include broad-spectrum antibacterial and antifungal agents. Cultures of the pus from the initial abscess evacuation tested positive for Streptococcus pneumoniae. Histopathological examination of samples taken during the second evacuation from the left superior temporal orbital tissue, left optic nerve sheath, and left lacrimal gland revealed fibroadipose tissue with acute inflammation and necrosis. Periodic acid-Schiff (PAS) and Grocott methenamine silver (GMS) stains were negative, and there was no evidence of malignancy.

## Discussion

Orbital cellulitis is a severe purulent inflammation with potential sight and life-threatening implications. Comprehensive patient history and meticulous serial examinations play pivotal roles in the management of this condition to determine its etiology, as addressing the underlying cause remains fundamental for effective treatment [2].

There are various etiologies of orbital cellulitis that have been identified. These include extensions from periorbital infections such as eyelid chalazion or hordeolum, adjacent dental infections, hematogenous dissemination from systemic infections, and post-traumatic manifestations [2]. Notably, a primary sinus infection is regarded as the predominant cause of orbital cellulitis [5]. Additionally, orbital cellulitis may arise as a complication from intraocular or periocular surgical procedures. For instance, a case was documented in 2016 in which a patient developed orbital cellulitis with multiple abscesses subsequent to strabismus surgery [6].

Clinical manifestations typically observed in patients with orbital cellulitis include eyelid swelling, dull and persistent orbital pain, erythema, tenderness, chemosis, restricted ocular motility, and proptosis [2,7,8]. In line with this, our case exhibited eyelid swelling, escalating pain, proptosis, diminished vision, and limited ocular movement, in conjunction with opacified bilateral paranasal sinuses indicative of orbital cellulitis secondary to paranasal sinusitis. Documented complications of orbital cellulitis include subperiosteal abscess, prolonged proptosis leading to corneal exposure and ulceration, optic neuropathy due to neuritis, panophthalmitis, and intracranial issues such as cavernous sinus thrombosis and meningitis [2,7]. Chandler et al. were the first to divide the illness into five phases, indicating the morbidity and severity of each (Table 3) [9]. In light of this, our case fits under group 4. Numerous retrospective studies and case series have indicated that the medial wall is the most frequent site of pus accumulation in orbital cellulitis. This predilection is attributed to the ethmoidal sinus’s fragile bone structure. While non-medial orbital cellulitis presentations are infrequent, they are deemed as potential sight and life-threatening conditions [2,5,7].

**Table 3:** Chandler's classification of the orbital cellulitis process

Chandler’s Classification		
Group 1	Preseptal cellulitis	Inflammation and edema anterior to the orbital septum.
Group 2	Orbital cellulitis	Extension of inflammation to include the orbital contents posterior to the septum
Group 3	Sub-periosteal abscess	Development of a mucopurulent collection between the bony orbital walls and periorbita
Group 4	Orbital abscess	Development of mucopurulent collection/s within the orbital contents
Group 5	Cavernous sinus thrombosis	Development of retrograde phlebitis and coagulation of vascular contents extending up to the cavernous sinus giving rise to bilateral ophthalmic deficits

In a study by Abtahi et al., it was found that 65.2% of patients with non-medial orbital cellulitis identified sinusitis and pansinusitis as the predominant sources of their condition, with the front-ethmoid sinuses being the most frequently affected [7]. In concordance with their findings, our case similarly exhibited superior collection coupled with bilateral pan-paranasal sinus involvement and opacification of the frontal sinus. Complications arising from sinusitis can be broadly categorized into local (osseous), orbital, and intracranial manifestations [10]. Intracranial complications stemming from sinusitis can escalate to life-threatening intracranial empyema and suppuration, presenting with symptoms of elevated intracranial pressure [11,12]. Orbital sequelae due to sinusitis range from preseptal cellulitis, and orbital cellulitis, to other related complications [10].

Orbital cellulitis is a clinical diagnosis; however, sino-orbital and brain imaging are required for the identification and confirmation of sinusitis, subperiosteal abscess, and/or periorbital abscess [2,13]. In our case, an enhanced orbital CT scan supplemented by thin-section CT imaging of the facial bones and sinuses revealed opacified pansinusitis and a superotemporal pus collection adjacent to the temporal aspect of the frontal sinus. In their study, Adedeji et al. delineated several determining factors in the choice of management: the severity of the disease, the progress despite medical intervention, the patient’s age, and the presence and management of complications [5]. Initial management of orbital cellulitis involves hospital admission, the prompt initiation of broad-spectrum antibiotics, and rigorous and regular assessments with an emphasis on evaluating optic nerve function. A retrospective analysis noted that for cases with concomitant sinusitis, endoscopic drainage surgery is required for medial and inferomedial collections. However, if the abscess is situated superiorly, an external drainage method is indicated [14]. Abtahi et al. documented a case in their retrospective evaluation in which a patient with superior and superotemporal collections underwent only external drainage surgery. Re-accumulation and worsening were noted three days post-surgery, mandating a combined surgical approach. This led to patient improvement without subsequent recurrence. Contrarily, our case, which presented with a superior collection resulting from pansinusitis, only underwent external drainage. This led to the need for another surgery in just a few days because of re-accumulation, symptom exacerbation, and eventual vision loss. Therefore, for non-medial collections, a combination of external abscess drainage and endoscopic sinus drainage is strongly recommended [7].

In the presented case, the pus culture obtained from the patient was positive for Streptococcus pneumoniae. It is notable that the predominant organisms isolated in cases of sinusitis-related orbital cellulitis in immunocompetent individuals include Staphylococcus aureus, various streptococcal species, Haemophilus influenzae, and anaerobes such as Bacteroides, Peptostreptococcus, and Fusobacterium [1,7].

Nwaorgu et al. have shown that the involvement of multiple sinuses indicates the disease’s severity, attributable to the continuous mucosal lining of the paranasal sinuses [1]. Additionally, Haemophilus influenzae and Streptococcus pneumoniae have been commonly identified in pre-vaccinated infants and children. It is also important to consider fungal pathogens and commensals such as Aspergillus, Rhizopus, Mucor, and Rhizomucor in immunocompromised individuals, given their potential to cause severe and life-threatening manifestations of this inflammatory condition [7,15].

Permanent visual loss is a devastating complication of orbital cellulitis. Three primary mechanisms for visual loss have been delineated: 1) optic neuritis arising from proximal inflammation, 2) occlusion of vessels nourishing the optic nerve, and 3) direct compression of the optic nerve due to pus accumulation [2]. In our case, the patient demonstrated pansinusitis that progressed to left orbital cellulitis, resulting in irreversible visual loss attributed to optic neuropathy. Concurrently, changes in the left orbital roof bone marrow were indicative of osteomyelitis.

## Conclusions

This report demonstrated a case of vision-threatening orbital cellulitis with the primary source of infection being sinus in origin. It was complicated by the formation of orbital and sub-periosteal abscesses. The patient’s condition worsened despite extensive investigations and the immediate initiation of empirical antibiotics on presentation. Additionally, multiple external abscess drainage was performed. However, the patient's condition progressed with no improvement and ended with a loss of vision and optic neuropathy. Hence, a high index of early combined surgical intervention and eradication of the primary source is important in cases of orbital cellulitis with non-medial abscesses formation.

In conclusion, the cornerstone of cellulitis management is the identification and treatment of its underlying cause. This is imperative to prevent severe complications such as irreversible vision loss secondary to optic neuropathy and potentially fatal cerebral venous thrombosis and meningitis accompanied by systemic sepsis. While non-medial abscesses are less common than medial abscesses, they are associated with a greater risk of vision- and life-threatening complications, as well as a high likelihood of re-accumulation. Therefore, prompt external drainage in conjunction with endoscopic sinus surgery is strongly recommended and advised.
